# Dither removing Fourier ptychographic microscope based on a two-axis rotation stage

**DOI:** 10.1117/1.JBO.26.3.036501

**Published:** 2021-03-04

**Authors:** Kaicheng Huang, Wangwei Hui, Qing Ye, Hongyang Zhao, Qiushuai Shi, Jianguo Tian, Wenyuan Zhou

**Affiliations:** Ministry of Education, Nankai University, School of Physics, Key Laboratory of Weak-Light Nonlinear Photonics, Tianjin, China

**Keywords:** Fourier ptychographic microscope, computational imaging, illuminator, dither removing algorithm

## Abstract

**Significance**: Large space-bandwidth product is highly desirable in many biomedical imaging. Fourier ptychographic microscopy (FPM) is a computational imaging technique that can significantly increase the space-bandwidth product of a standard microscope. The illuminator of a Fourier ptychographic microscope is not flexible at present, and it is inconvenient to meet different imaging needs.

**Aim**: An illuminator based on a two-axis motorized rotation stage was presented to provide a more flexible illuminating way with the goal of meeting different imaging needs.

**Approach**: The illuminator adopts a concentric illuminating method to provide coherent illumination in any direction on the sample plane. The sampling pattern can be freely designed and changed according to the parameters of the imaging system. A dither removing algorithm was proposed to remove the potential dither influence introduced in the image acquisition process.

**Results**: The illuminator could be conveniently integrated into different imaging systems. The feasibility and flexibility were demonstrated by applying it to imaging systems with numerical aperture of 0.045 and 0.01. The resolution gain is about 4- and 13-fold, respectively. The effectiveness of the dither removing algorithm was validated in both simulation and experiment.

**Conclusions**: A more flexible illuminator for FPM was presented to meet different imaging needs. A dither removing algorithm was proposed to remove dither influence.

## 1 Introduction

Fourier ptychographic microscopy (FPM) is a recent computational imaging technique that is capable of increasing the resolution of a standard microscope without sacrificing the field of view (FOV).^1^^–^^4^ By applying a phase retrieval algorithm, FPM stitches a series of low-resolution images to produce a high-resolution image.^5^^,^^6^ In addition, the phase of the reconstructed high-resolution image can be recovered without knowing the phases of the low-resolution images.^4^ FPM has made great progress in many aspects, such as apparatus structure,^7^^–^^11^ experimental method,^12^^–^^21^ parameter correction,^3^^,^^22^^–^^24^ and reconstruction algorithm.^25^^–^^28^

So far, most FPM platforms are using a light-emitting diode (LED) array as an illuminator. An LED array can provide stable and fast illumination without mechanical movement. However, an LED array has its own drawbacks as an illuminator in FPM. First, the intensity of an LED is relatively weak, especially for large-angle illumination. Second, the distribution density of LEDs is fixed, which is inflexible for adjusting the sampling density in spectrum. Third, the largest illumination angle is limited, which is inflexible for synthesizing a very large NA. Fourth, the positions of all LEDs are fixed; therefore, the sampling patterns with an LED array are limited. These drawbacks of an LED array lead to the emergence of some different illuminators in FPM. Chung et al.^9^ have developed an illuminator using laser source guided by a galvo-scanner and a mirror array and achieved fast acquisition, but it is inflexible for the mirror array to change the sampling pattern. Guo et al.^29^ have developed a flexible illuminator using liquid crystal display, the illuminator is convenient to change the sampling pattern but is inconvenient for large incident angles since the maximum incident angle is limited by the condenser. There are other different illuminators, such as quasi-dome LED array,^30^ hemispherical condenser,^10^^,^^31^ and illuminator with digital micromirror device.^8^^,^^32^ These illuminators are still inflexible in some aspects; therefore, they could not be conveniently utilized to investigate the FPM technique under different conditions.

To provide a more convenient way to investigate FPM technique in depth, we present a more flexible illuminator for FPM. The illuminator uses a laser as its illumination source mounted on a two-axis motorized rotation stage. We adopt a concentric illuminating method, that is, the laser always illuminates the intersection of the two axes. The illuminator is capable of providing coherent illumination in any direction with an angular resolution of 0.0003125 deg and a maximum incident angle of 90 deg. Therefore, it is easy to design and change the sampling pattern in the FPM with a very high degree of freedom. The illuminator can be easily integrated into different FPM platforms with different parameters. It is quite convenient to use this illuminator to investigate various deep-seated problems related to FPM.

To reconstruct high-resolution image, FPM needs to capture a series of low-resolution images. Keeping the acquisition process stable is essential for reconstructing high-quality image. Our illuminator is driven in mechanical way, which inevitably introduces little dither during the FPM acquisition process. One feasible approach is to wait until the platform is stable before each image acquisition. However, this approach would greatly increase the acquisition time. To obtain the next image quickly without being disturbed by dither, we propose a dither removing algorithm for FPM. The dither removing algorithm is able to reconstruct a high-resolution image successfully from a series of low-resolution images polluted by dither. With the dither removing algorithm, the stability requirement can be relaxed and the acquisition time can be shortened.

The remainder of this paper is structured as follows. In Sec. 2, we describe the schematic of the setup that we used in the experiments and the principle of the dither removing algorithm. In Sec. 3, we first verify the effectiveness of our proposed dither removing algorithm by simulation. Then we perform three experiments: The first experiment demonstrates the feasibility of our illuminator and the effectiveness of our proposed dither removing algorithm. The second experiment shows the flexibility and wide applicability of the illuminator. The last experiment shows that the illuminator is applicable for real biological specimens. In Sec. 4, we present the conclusions of our works.

## 2 Method

### 2.1 Experimental Setup

**Fig. 1:**
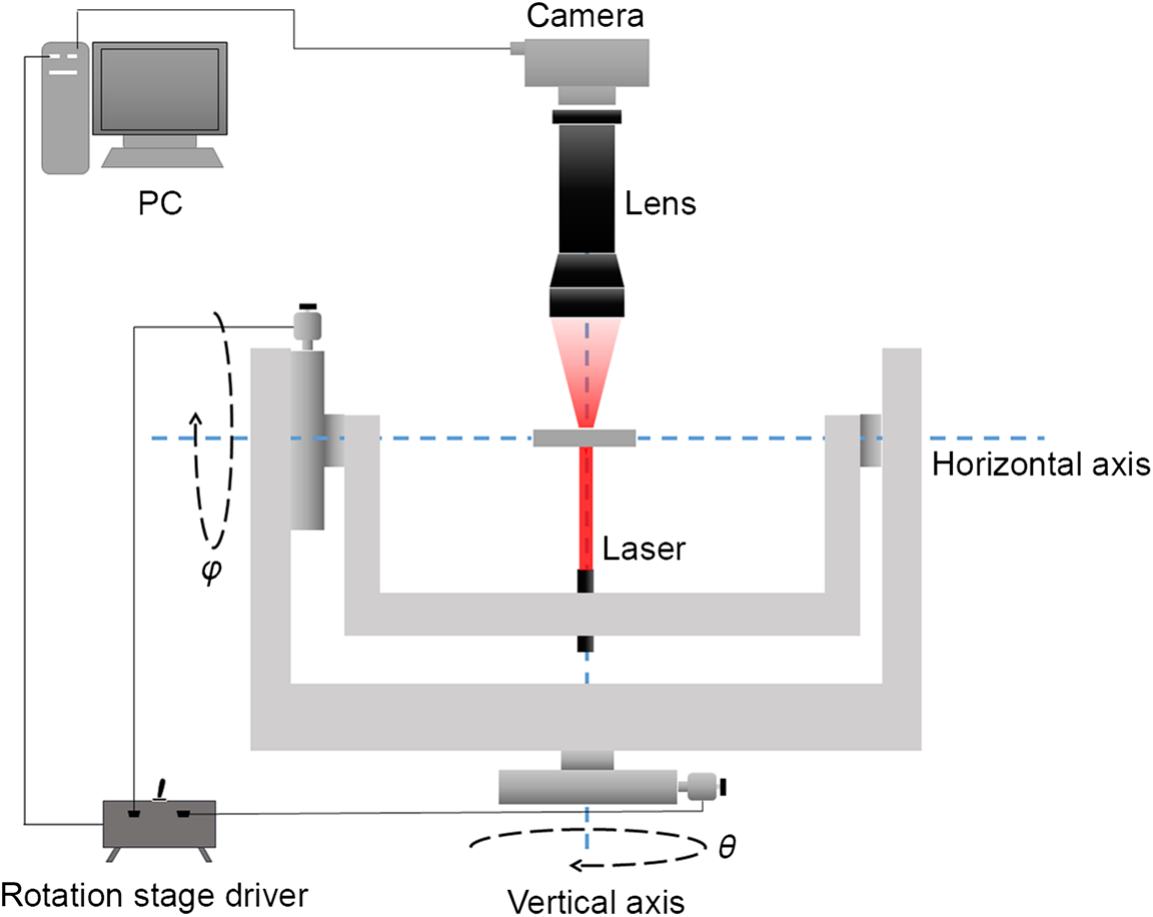
Schematic of the imaging platform.

We integrate our illuminator into an FPM platform, which is described as follows. The illumination source is a laser whose wavelength is 635 nm. The laser is mounted on a two-axis motorized rotation stage. We adopt a concentric illuminating method, that is, the laser always illuminates the intersection of the two axes. We denote the angle associated with the vertical axis as θ and the angle associated with the horizontal axis as φ. θ and φ can cover the whole spatial angle with an accuracy of 0.0003125 deg. We adjust the laser beam to coincide with the vertical axis at the beginning. Benefitting from the flexibility of the rotation stage, the sampling pattern can be freely designed and changed according to the system parameters, making the illuminator widely applicable. The specimen is placed at the intersection of the two axes. The imaging lens is a 1× telecentric lens (WWH10-110CT-G, Coolens), whose numerical aperture (NA) is adjustable in the range of 0.005 to 0.045. A complementary metal-oxide-semiconductor camera is used with a pixel size of 3.45  μm (GS3-U3-123S6M-C, FLIR). The structure of the imaging platform is shown in Fig. 1.

### 2.2 Dither Removing Algorithm

In a general microscope imaging platform, the pixel size on the object plane can be calculated by d/mag, which is about a few hundred nanometers to a few microns, where d is the pixel size of the camera and mag is the magnification of the system. It is inevitable to introduce little dither especially when starting and stopping mechanical movement. In FPM, the presence of dither will degrade the quality of the reconstructed image. It becomes especially essential to find the unknown individual shifted value of each low-resolution image correctly. Bian et al.^23^ have tackled the sample motion problem using an annealing method. However, the dither in our application is relatively small, so here we use a more lightweight method to solve the dither problem. The main idea of our algorithm is straightforward. For each illumination direction, after updating the spectrum in conventional FPM recovery procedure,^1^ we use this spectrum to correct the dither value of next image. We simulate the imaging process of the corresponding illumination direction. We assume that the captured image has the identical distribution as the simulated image except for a lateral shift. We apply the phase correlation algorithm to find the shifted value of the captured image^33^^,^^34^ and use the found shifted value to shift the captured image back. Then the square root of the dither removed image is used to substitute the amplitude of the simulated image. The algorithm is described in detail below.

As suggested by the FPM recovery algorithm,^1^ we initialize an estimation of the high-resolution spatial spectrum $\tilde{O}(f)$ first, where f represents the spatial frequency coordinate. Since the estimated spectrum could be different from the true spectrum, if necessary, we could perform a few iterations of the conventional FPM recovery procedure (experiments show that two iterations are sufficient). Then we calculate the simulated low-resolution image $I_{i}^{s}(r)$ under illumination of the i’th direction,^22^

$$I_{i}^{s}(r)={\left|F^{-1}\{\overset{˜}{O}(f-f_{i})\cdot \overset{˜}{P}(f)\}\right|}^{2},$$ (1)

where r represents the spatial coordinate, $F^{-1}$ represents the inverse Fourier transform, $f_{i}$ is the illumination wavenumber of the i’th image, and $\tilde{P}(f)$ represents the pupil function. Let $I_{i}(r)$ denote the recorded image under illumination of the i’th direction. Under our assumption, we have

$$I_{i}^{s}(r)=\alpha_{i}I_{i}(r+r_{i}),$$ (2)

where $\alpha_{i}$ is a constant factor and $r_{i}$ is the unknown i’th shifted value introduced by dither. If we can find the exact value of $r_{i}$, then we can remove the influence of dither by simply replacing $I_{i}(r)$ with $I_{i}(r+r_{i})$. When we calculate the Fourier transform on both sides of Eq. (2), we have

$${\overset{˜}{I}}_{i}^{s}(f)=\alpha_{i}{\overset{˜}{I}}_{i}(f)e^{j2\pi f\cdot r_{i}},$$ (3)

where ${\overset{˜}{I}}_{i}^{s}(f)$ represents the Fourier transform of $I_{i}^{s}(r)$ and ${\overset{˜}{I}}_{i}(f)$ represents the Fourier transform of $I_{i}(r)$. We multiply both sides of Eq. (3) by ${\overset{˜}{I}}_{i}^{*}(f)/{\left|{\overset{˜}{I}}_{i}(f)\right|}^{2}$ and calculate the inverse Fourier transform, and we mark it as $D_{i}(r)$,

$$D_{i}(r)=F^{-1}\{\frac{{\overset{˜}{I}}_{i}^{s}(f){\overset{˜}{I}}_{i}^{*}(f)}{{\left|{\overset{˜}{I}}_{i}(f)\right|}^{2}}\}=\alpha_{i}F^{-1}\{e^{j2\pi f\cdot r_{i}}\}=\alpha_{i}\delta (r+r_{i}),$$ (4)

where δ(r) is the Dirac delta function. We determine $r_{i}$ by

$$r_{i}=-\underset{r}{\arg\;\max}|D_{i}(r)|.$$ (5)

**Fig. 2:**
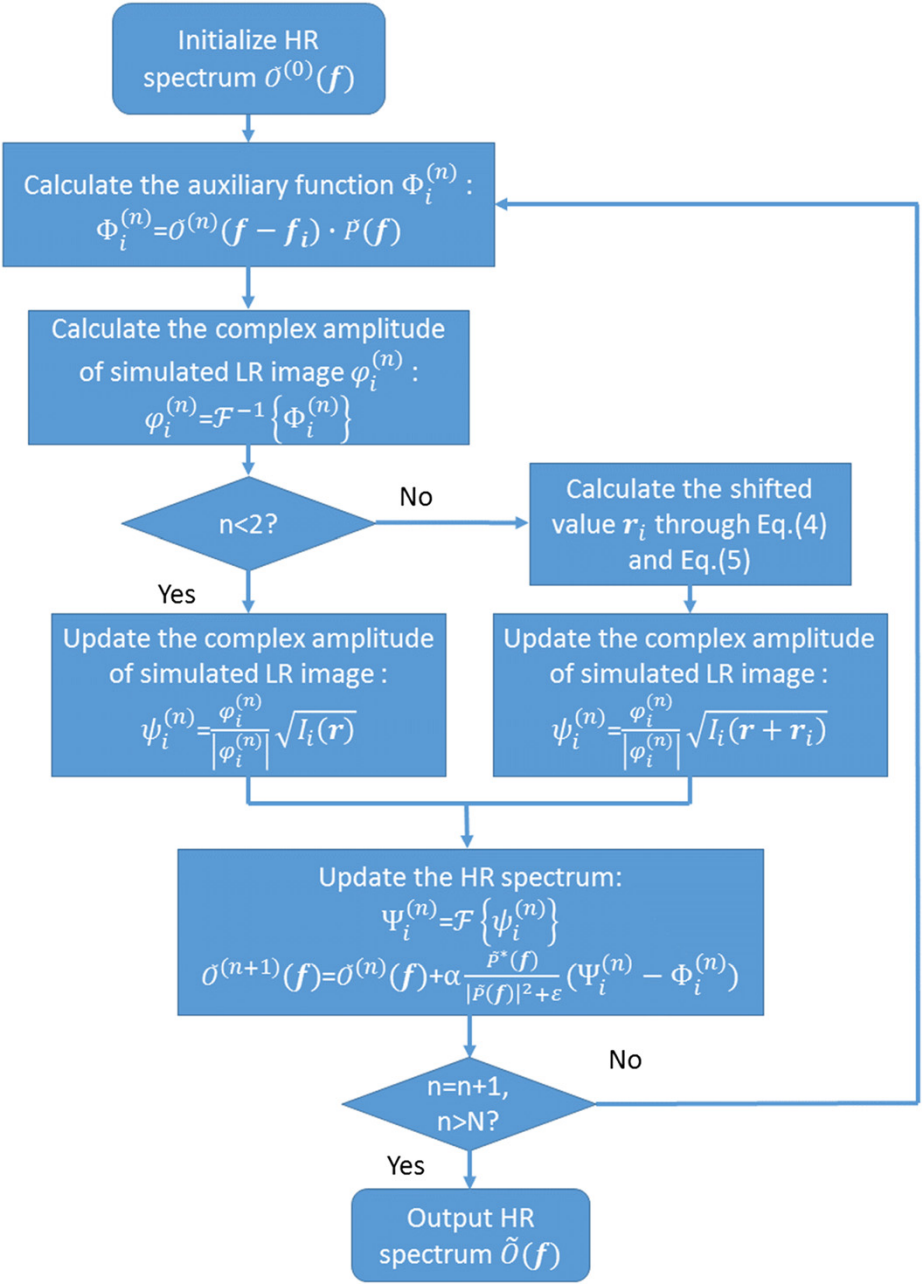
Flowchart of the dither removing algorithm.

In practice, Eq. (2) usually does not hold, nor is $D_{i}(r)$ a shifted Dirac function. However, as long as $I_{i}^{s}(r)$ is similar with $I_{i}(r)$, $D_{i}(r)$ is a unimodal function, and Eq. (5) still works. We will not prove it analytically, as it will be demonstrated in simulation and experiments later. Then $I_{i}(r)$ is replaced with $I_{i}(r+r_{i})$ to participate in the FPM recovery procedure. The flowchart of the dither removing algorithm is summarized in Fig. 2.

### 2.3 Dither Calibration

The dither removing algorithm we proposed does not consider the motion blur problem. To theoretically analyze the feasibility of the dither removing algorithm without considering the motion blur, we calibrated the dither of our experimental setup. Then we gave a sufficient condition that does not need to consider the motion blur.

**Fig. 3:**
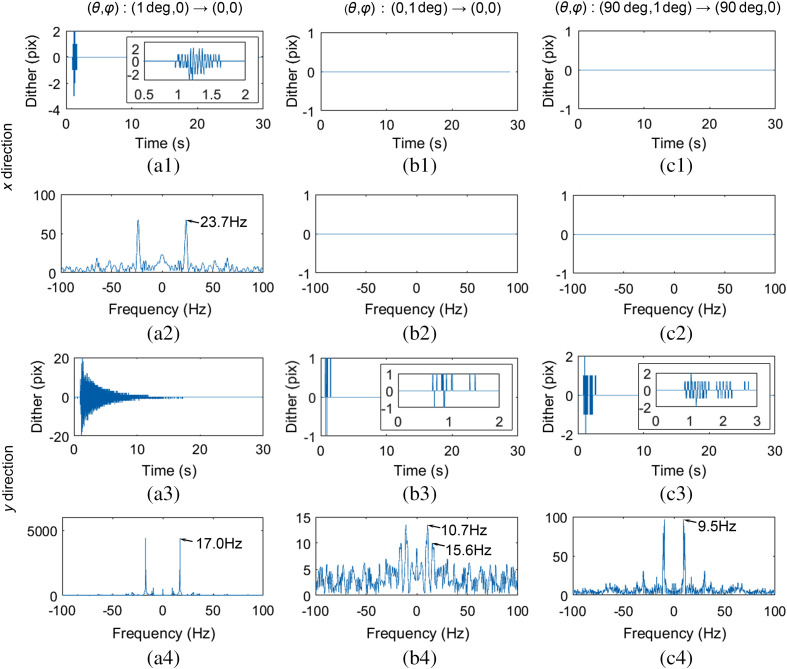
Dither calibration results. (a1) The dither of the first type of movements in x direction over time, (a2) the frequency spectrum of (a1), (a3) and (a4) have similar meanings to (a1) and (a2) but in y direction. (b1)–(b4) and (c1)–(c4) are similar to (a1)–(a4) but belong to the second and third types of movements.

The method that we calibrate the dither is described as follows. First, we start to capture images of the sample in a subregion of 352×360  pixels in static state at a framerate of 200 fps. Immediately we rotate the stage by an angle. We keep capturing images for about 30 s. When the image acquisition process is finished, we use the first image as the standard and calculate the shifted values for the remaining images using phase correlation method.^33^^,^^34^ We calibrated the dither for three types of movements: (1) (θ,φ) from (1 deg, 0) to (0, 0); (2) (θ,φ) from (0, 1 deg) to (0, 0); and (3). (θ,φ) from (90 deg, 1 deg) to (90 deg, 0). The angular speed of the rotation stage is set to be 8 deg/s. The calibration results are plotted in Fig. 3.

Figure 3(a1) shows the dither of the first type of movements in x direction over time. Figure 3(a2) shows the frequency spectrum of Fig. 3(a1). Figures 3(a3) and 3(a4) have similar meanings to Figs. 3(a1) and 3(a2) but in y direction. Figures 3(b1)–3(b4) and 3(c1)–3(c4) are similar to Figs. 3(a1)–3(a4) but belong to the second and third types of movements. Comparing the results of the three types of movements, we can conclude that the dither is more severe when rotating the stage along the vertical axis than along the horizontal axis. Comparing the results in x and y directions, we can conclude that the dither is more severe in y direction than in x direction. This conclusion can be also obtained by comparing the results of the last two types of movements since the dither is a little more severe when θ is 90 deg than 0. Results in Fig. 3 show that the dominant frequency when rotating the stage along the vertical axis is about 20 Hz and the dominant frequency when rotating the stage along the horizontal axis is about 10 Hz.

**Fig. 4:**
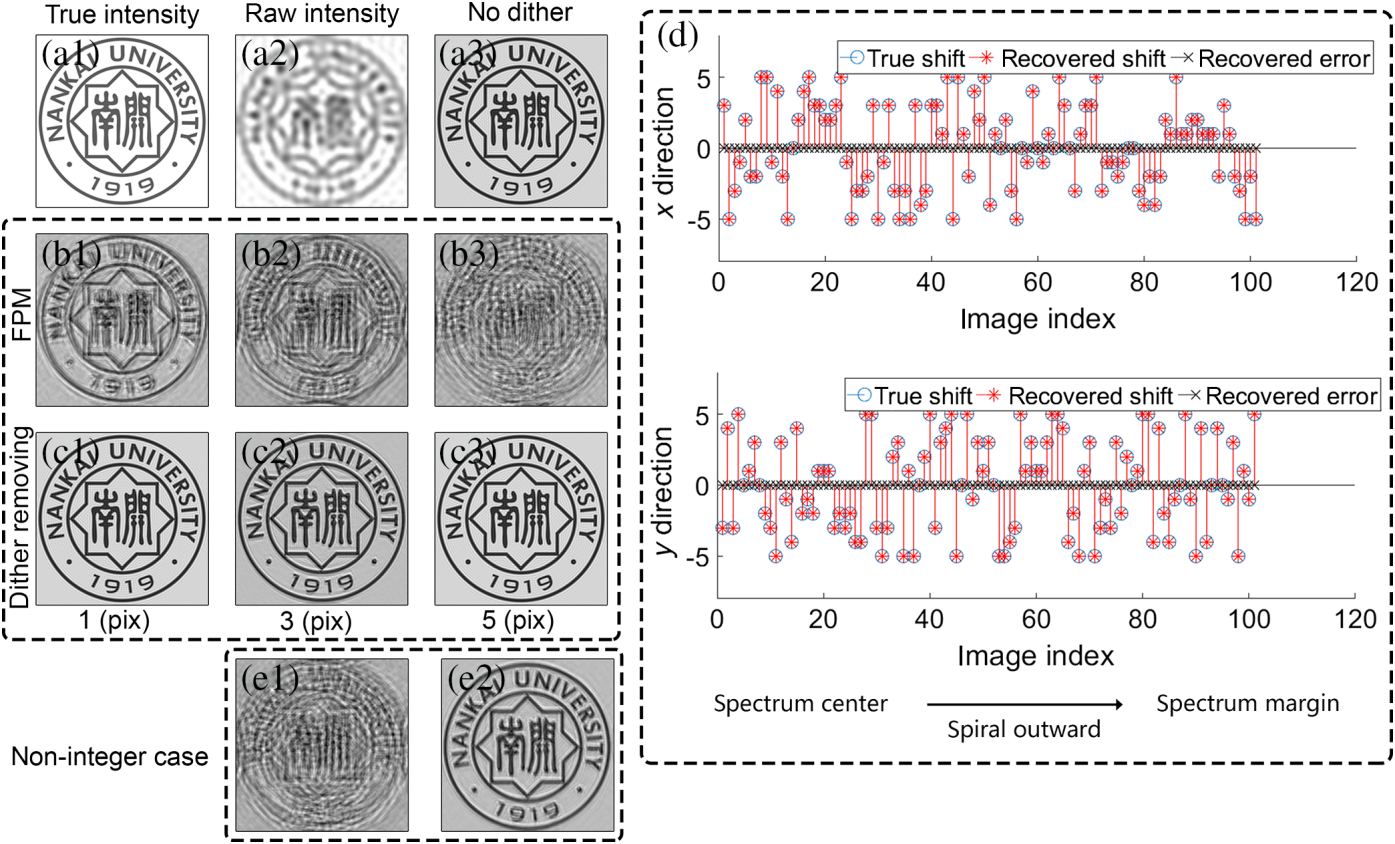
Simulation results. (a1) The true image intensity of the simulated object, (a2) the raw image under normal illumination, and (a3) the reconstruction intensity of the traditional FPM algorithm applied to the dither-free dataset. (b1)–(b3) The intensities recovered by the traditional FPM algorithm whose maximum shifted pixels are 1, 3, and 5. (c1)–(c3) Similar with (b1)–(b3) except that they are reconstructed by the dither removing algorithm. The true and recovered shifted values are plotted in (d). (e1) and (e2) The reconstructed intensities without and with the dither removing algorithm of non-integer case, respectively.

It is reasonable to use simple harmonic vibration model to represent the dither in a short time duration (T to T+ΔT). We represent the dither D as

$$D(t)=N_{T}\;\sin(2\pi ft),\;T\leq t\leq T+\Delta T,$$ (6)

where $N_{T}$ is the amplitude in pixel around time T and f is the dominant frequency. The maximum velocity can be expressed as $2\pi fN_{T}$. When we capture an image, we think the motion blur can be neglected if the image travels less than 1 pixel. If the exposure time is Δt, we give a sufficient condition for not considering the motion blur by Eq. (7),

$$2\pi fN_{T}\Delta t<1.$$ (7)

For rotating the stage along the horizontal axis, most $N_{T}$ is no more than 1 pixel, f is about 10 Hz, we can deduce that if Δt≤16  ms, we can neglect the motion blur. For rotating the stage along the vertical axis, the case will be worse. f is about 20 Hz. If $N_{T}$ is 20, Δt should be less than 0.4 ms, and if $N_{T}$ is 5, Δt should be less than 1.6 ms.

In practice, we rotate the stage along the vertical axis to a specified θ, and then rotate the stage along the horizontal axis to all φs we needed. So the number of rotations along vertical axis is much less than that along horizontal axis. When changing θ, we wait for 3 s before capturing images to reduce the $N_{T}$ to about 5. The exposure time of bright-field images is set to be 0.6 ms. We increase the exposure time as φ goes up until the exposure time reaches 19.2 ms. Then we increase the gain instead if φ continues to go up. (But we cannot increase the gain too much since it will bring in noise.) In this way, we can ensure that most of the images are free of motion blur, and images that could be possibly polluted with motion blur correspond to the largest angles. This strategy works well in our experiments.

## 3 Results

### 3.1 Simulation

We first demonstrate the effectiveness of the dither removing algorithm in simulation. We generate a dither-polluted simulation dataset by artificially adding a random shifted value to each simulated image. Concretely, in the process of simulating image acquisition, we artificially add the influence of dither for the i’th image by shifting the image with $\left(\Delta x_{i},\Delta y_{i}\right)$ pixels, where $\Delta x_{i}$ and $\Delta y_{i}$ are independent discrete random variables and have the identical probability distribution:

$$P(\Delta x_{i}=\Delta )=P(\Delta y_{i}=\Delta )=\{\begin{array}{ll} \frac{1}{2p+1}, & \text{if }\Delta =-p,-p+1,\ldots ,p-1,p\\ 0, & \text{otherwise} \end{array},$$ (8)

where p>0 is a preset positive integer that indicates the maximum shifted pixels due to dither. We simulate the situation where p is 1, 3, and 5. We compare the reconstruction results of the dither removing algorithm and the traditional FPM algorithm. The reconstruction results of simulation are summarized in Fig. 4.

Figure 4(a1) shows the true image intensity of the simulated object, Fig. 4(a2) shows the raw image under normal illumination, and Fig. 4(a3) shows the reconstruction intensity of the traditional FPM algorithm applied to the dither-free dataset. Group (b) and (c) in Fig. 4 are the results reconstructed from the dataset polluted by dither. Figures 4(b1)–4(b3) show the intensities recovered by the traditional FPM algorithm whose maximum shifted pixels are 1, 3, and 5 while Figs. 4(c1)–4(c3) show the intensities recovered by the dither removing algorithm whose maximum shifted pixels are 1, 3, and 5. Results of group (b) in Fig. 4 show that dither can severely degrade the reconstruction quality. Results of group (c) in Fig. 4 show that the dither removing algorithm can effectively remove the influence of dither and successfully recover the image.

It is worth noting that the reconstructed image is allowed to have an uncertain global shifted value. Suppose H(r) is the reconstructed image through the dither removing algorithm and $\left\{r_{i}|i=1,2,\ldots ,n\right\}$ is the shifted value set of the image set, n is the total number of raw images. It is easy to understand that $H(r+r_{0})$ could also be a reconstructed image with the shifted value set $\left\{r_{i}+r_{0}|i=1,2,\ldots ,n\right\}$, where the Euclidean norm of $r_{0}$ is much smaller than the range of the image. In practice, when the high-resolution image H(r) is reconstructed, we shift the image by $\overset{¯}{r}$, i.e., $H(r-\overset{¯}{r})$, where $\overset{¯}{r}=\overset{n}{\underset{i=1}{\sum }}r_{i}/n$. And the corresponding shifted value set would be $\left\{r_{i}-\overset{¯}{r}|i=1,2,\ldots ,n\right\}$, which is zero mean. So $H(r-\overset{¯}{r})$ would not shift too far from the true image since the dither value is also zero mean.

We also examine the recovered shifted values with the case p is 5. The result is plotted in Fig. 4(d). The blue circles represent the true shifted values while the red asterisks represent the recovered shifted values. The black crosses represent the recovered errors. The image with index 1 is corresponding to the spectrum center. The position in spectrum goes outward in a spiral way with an increasing image index. Figure 4(d) shows that in this case, all images retrieved the correct shifted values. Figure 4(d) demonstrates that the recovered shifted value by dither removing algorithm is quite reliable.

The shifted values artificially added to the image sequence by Eq. (8) are all integers. In practice, the shifted values cannot be exactly integers. To examine the recovery quality in non-integer cases, we simulate the non-integer dither by interpolation. First, we upsample all the captured images by 10 times. Then we add the influence of dither as before, where p is 50. Finally, we downsample all images to their original size. In thus constructed images, the precision of shifted values is 0.1 pixel and the maximum shifted value is 5. Figures 4(e1) and 4(e2) show the reconstructed intensities without and with the dither removing algorithm. Results demonstrate that the dither removing algorithm is also effective in non-integer cases.

### 3.2 Experiments

To verify the feasibility of the illuminator and the effectiveness of the dither removing algorithm in realistic imaging, we conduct an experiment on the imaging platform using the dither removing algorithm. In this experiment, we adjust the NA of the lens to the maximum value of 0.045. We use a non-uniform sampling pattern for better sampling efficiency and image quality.^13^ We set $\Delta \varphi =\Delta \varphi_{1}$ when $\varphi \leq \varphi_{\text{threshold}}$ and $\Delta \varphi =\Delta \varphi_{2}$ when $\varphi_{\text{threshold}}\leq \varphi \leq \varphi_{\max}$ (the value of Δφ should meet the overlapping condition).^35^ We adopt a simple strategy to design the sampling pattern:$\varphi_{0}=0$, for $\varphi_{n}=\varphi_{n-1}+\Delta \varphi \leq \varphi_{\max}$, we set θ to be evenly distributed on the circumference. When n is 0, we only sample one angle where θ is 0. When n is 1, we sample four angles where θ is 0 deg, 90 deg, 180 deg, and 270 deg, respectively. Otherwise, we temporarily set the number of θ to be the same as in the case of n−1. If the adjacent subareas of the spectrum do not overlap with each other, we double the number of θ. Here, we set $\Delta \varphi_{1}=1\;\deg$, $\Delta \varphi_{2}=2\;\deg$, $\varphi_{\text{threshold}}=4\;\deg$, and $\varphi_{\max}=8\;\deg$. The total number of sampling angles is 45. The maximum exposure time is 5.4 ms with gain of 0.

**Fig. 5:**
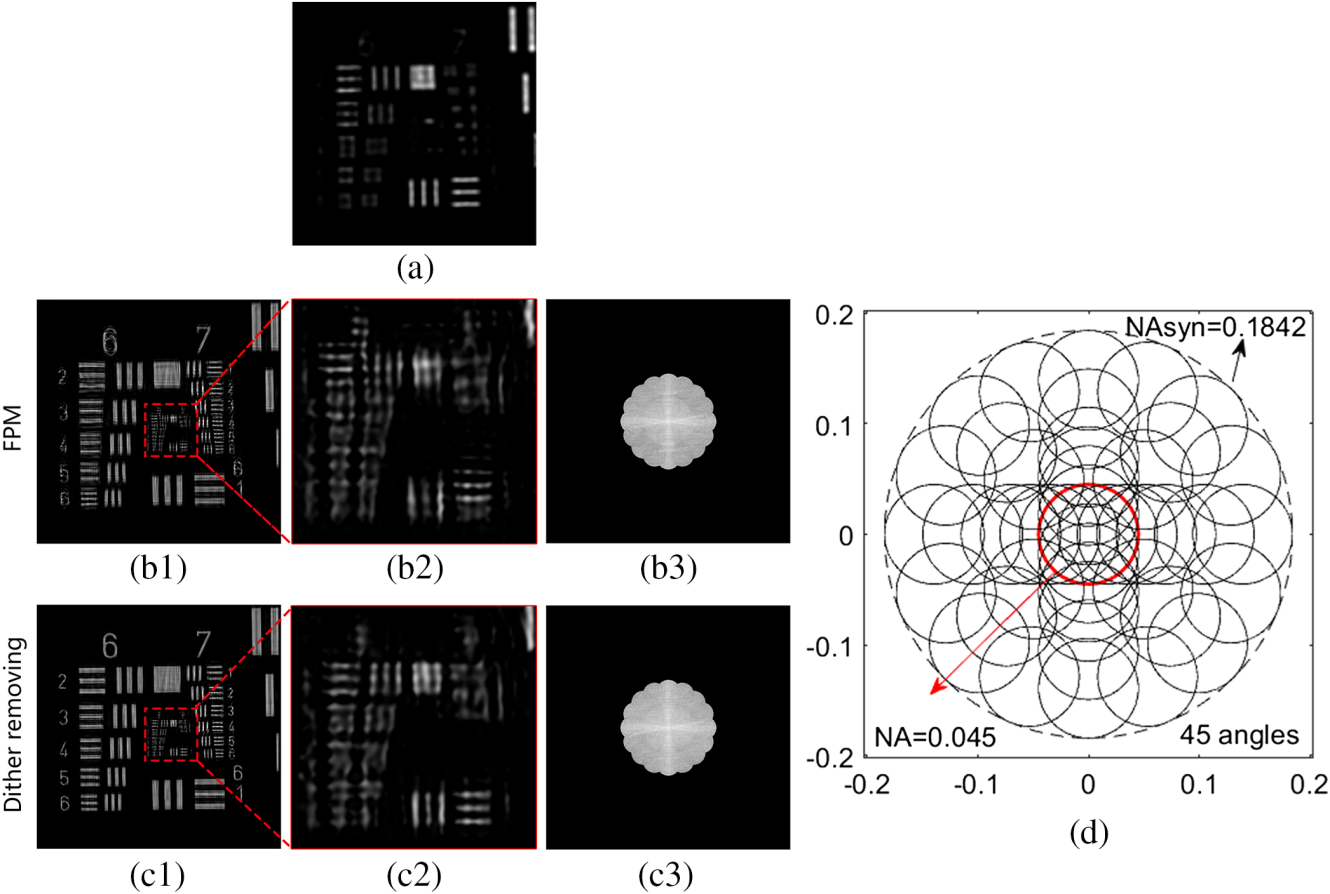
Experimental comparison results. (a) The raw image; (b1) and (c1) the intensities reconstructed by FPM without and with dither removing, respectively; (b2) and (c2) zoomed-in subarea of (b1) and (c1), respectively. (b3) and (c3) The spectra reconstructed by FPM without and with dither removing, respectively. (d) The designed sampling pattern.

The sample in this experiment is a USAF resolution target. We compare the reconstruction results of the dither removing algorithm and the traditional FPM algorithm. The experimental results are summarized in Fig. 5.

Figures 5(a) shows the raw image. Figures 5(b1) and 5(c1) show the intensities reconstructed by FPM without and with dither removing, respectively. Figures 5(b2) and 5(c2) show a zoomed-in subarea of Figs. 5(b1) and 5(c1), respectively. Figures 5(b3) and 5(c3) show the spectra of the complex amplitudes of Figs. 5(b1) and 5(c1), respectively. Figures 5(b1) and 5(b2) show that the quality degration of the reconstructed image is more severe in y direction than in x direction. It is consistant with the calibration results in Sec. 2.3. Figures 5(c1) and 5(c2) indicate that the quality of reconstructed image can be improved by dither removing algorithm. The sampling pattern in this experiment is portrayed in Fig. 5(d). The system NA is 0.045 (the red circle) and the theoretical synthetic NA is 0.1842 (the big dashed black circle), and the effective resolution gain is about 4-fold. This experiment verifies the feasibility of our illuminator and the effectiveness of the dither removing algorithm. The application of non-uniform sampling pattern also shows the incident angle selection flexibility of our illuminator.

**Fig. 6:**
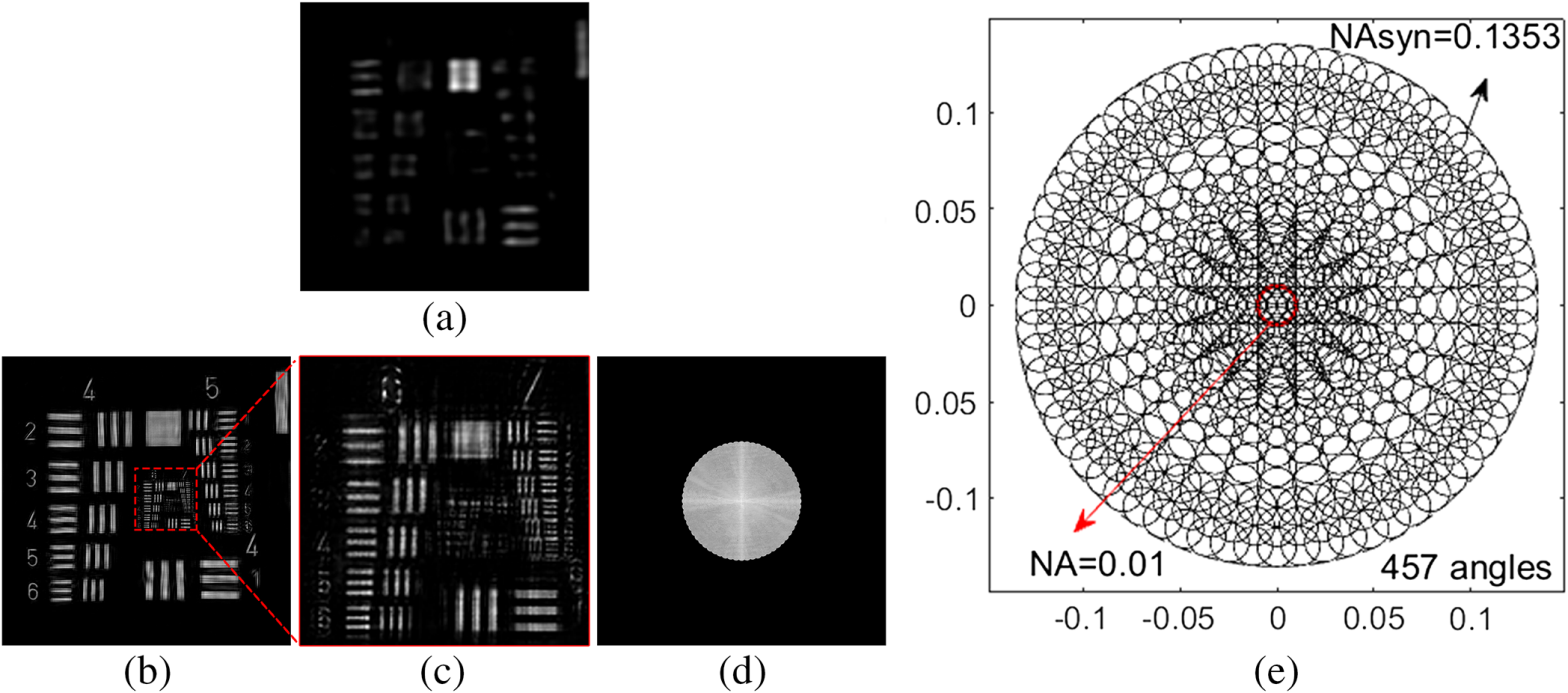
Experimental results with very low NA. (a) The raw image, (b) the reconstructed image of (a), (c) the zoomed-in subarea of the red dashed square in (b), (d) the reconstructed spectrum, and (e) the designed sampling pattern.

Then we apply the imaging platform to an experiment with very low system NA to show the flexibility and wide applicability of the illuminator. In this experiment, we adopt the same strategy with the first experiment to design the sampling pattern. We set NA to be 0.01, $\Delta \varphi_{1}=0.3\;\deg$, $\Delta \varphi_{2}=0.6\;\deg$, $\varphi_{\text{threshold}}=3\;\deg$, and $\varphi_{\max}=7.2\;\deg$. The total number of sampling angles is 457. The maximum exposure time is 19.2 ms with gain of 12.11. The experimental result is shown in Fig. 6.

Figure 6(a) shows the raw image, Fig. 6(b) shows the reconstructed intensity, and Fig. 6(c) shows the zoomed-in subarea of the red dashed square in Fig. 6(b). Figure 6(d) shows the reconstructed spectrum. The sampling pattern is portrayed in Fig. 6(e). The system NA is 0.01 (the red circle) and the theoretical synthetic NA is 0.1353 (the big dashed black circle), and the effective resolution gain is about 13-fold. Figure 6 shows that our imaging platform is also applicable to a very low-NA objective lens. It demonstrated that our illuminator is quite flexible and widely applicable.

**Fig. 7:**
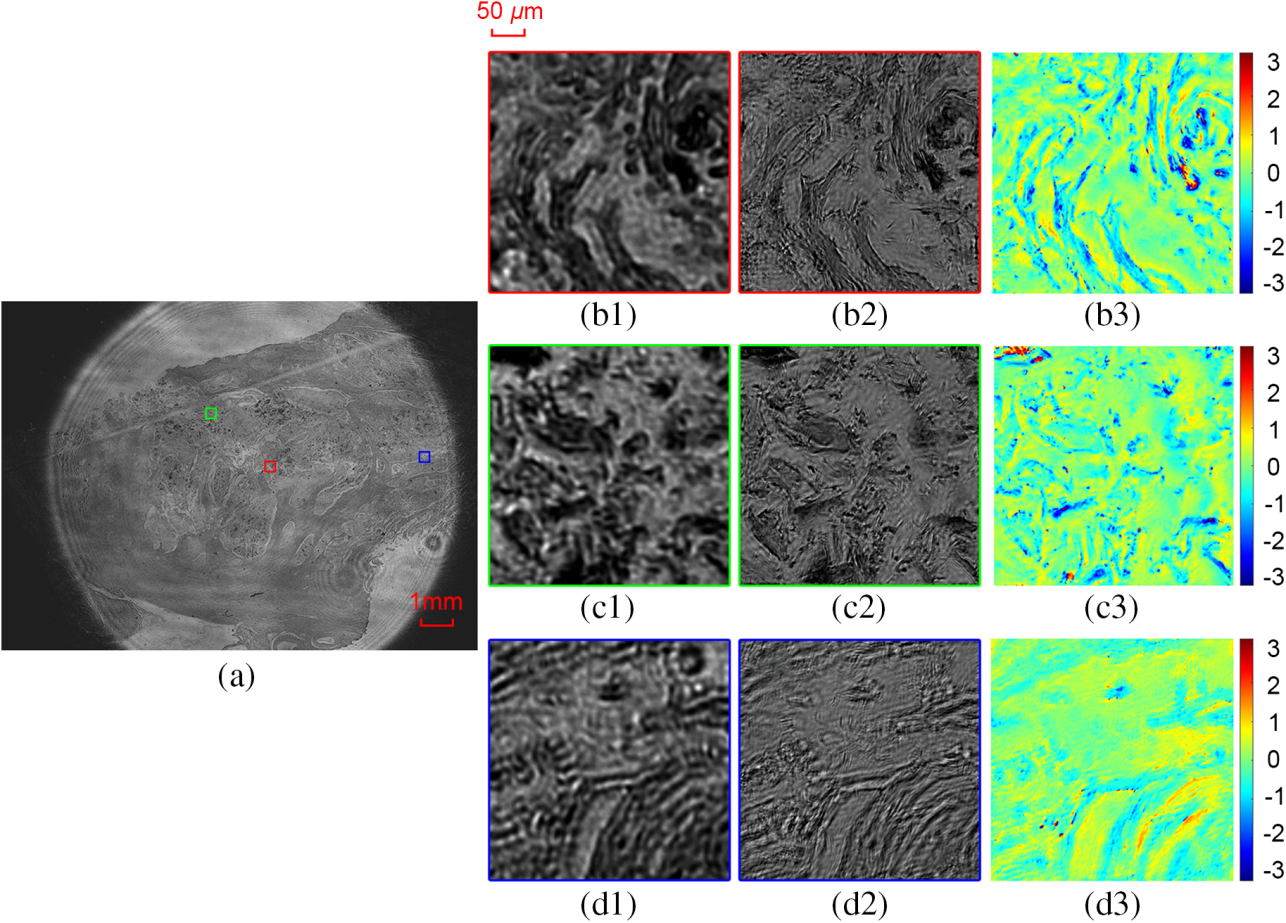
Experimental results of well-differentiated squamous cell carcinoma. (a) The FOV raw image, (b1)–(d1) three zoomed-in subareas of the squares in (a), (b2)–(d2) the reconstructed intensities, and (b3)–(d3) the reconstructed phases.

To demonstrate that the illuminator is also feasible for a real biological specimen, we apply the imaging platform to well-differentiated squamous cell carcinoma. We use the same parameters and sampling pattern as the first experiment. Figure 7 shows the experimental results. Figure 7(a) shows the FOV image. Figures 7(b1)–7(d1) show the zoomed-in subareas of different squares in Fig. 7(a). Figures 7(b2)–7(d2) and 7(b3)–7(d3) show the reconstructed intensities and phases of (b1)-(d1), respectively. Results demonstrated that our platform is also applicable for a real biological specimen.

## 4 Conclusion and Discussion

In conclusion, we have presented a quite flexible illuminator for FPM based on a two-axis motorized rotation stage. It is capable of providing coherent illumination in any direction; therefore, the sampling pattern in FPM can be freely designed and changed. This illuminator provides a quite convenient way for investigating FPM in a deeper level and a wider range. To prevent tiny dither introduced by mechanical movements from degrading the reconstruction quality, we proposed a dither removing algorithm for FPM. The dither removing algorithm is able to retrieve a high-resolution image successfully from a sequence of low-resolution images polluted by dither. Simulation and experiment have verified the feasibility of our illuminator as well as the effectiveness of our dither removing algorithm. We demonstrated the high flexibility of our illuminator by applying it in FPM experiments with a 0.045 NA lens as well as a 0.01 NA lens and achieved a resolution gain of 4- and 13-fold, respectively.

A large FOV is very appealing in pathological section. However, a large FOV usually means a low NA, which requires illumination with a much denser angular distribution. Our illuminator can be integrated into a low-NA FPM platform conveniently. Our illuminator is quite suitable for investigating extremely high-resolution gain FPM, such as from 0.04 to 0.8 or more.

We use a laser as the illumination source, which is sensitive to any optical imperfection. It will bring fluctuating background signals and speckles in the captured images, which could be seen in Fig. 7(a). These fluctuations could then contribute negatively to the reconstructed phase. For a thin sample (with small phase), one can use the differential phase contrast deconvolution in the FP algorithm to further improve the quality of reconstructed phase.^9^ We will use a more appropriate illumination source to further improve the reconstructed complex image in the future.

In many other cases, the motion blur problem cannot be neglected. We will investigate the motion blur problem and improve the dither removing algorithm in the future.

Due to the mechanical scanning, the slow acquisition speed is a disadvantage of our illuminator. So it is not suitable for the cases that have high requirement for time.

We believe that our illuminator and the dither removing algorithm will help the application and theory investigation for FPM.
